# Development of a patient reported outcome instrument for chronic sialadenitis

**DOI:** 10.1186/s40463-022-00555-z

**Published:** 2022-02-04

**Authors:** Fatemeh Ramazani, Amr Hamour, Caroline C. Jeffery, Vincent Biron, Yaser Alrajhi, Daniel O’Connell, David W. J. Côté

**Affiliations:** Division of Otolaryngology-Head and Neck Surgery, Department of Surgery, 1E4 Walter C. MacKenzie Health Sciences Centre, University of Alberta, Edmonton, AB T6G 2B7 Canada

**Keywords:** Sialendoscopy, Sialadenitis, Patient reported outcomes

## Abstract

**Background:**

Sialendoscopy assisted treatments are a minimally invasive management modality for chronic sialadenitis. Clinicians report improved patient quality of life (QoL) following sialendoscopy assisted treatments, but there exist gaps in current literature about patient reported outcomes (PROs). PROs are outcome measures developed based on patient perceptions.

**Objective:**

The objective of this study was to create a PRO instrument for chronic sialadenitis, to assess the efficacy of sialendoscopy assisted treatments in improve patients’ QoL.

**Design:**

This four-phase qualitative study employed grounded theory methodology and a modified Delphi technique. In Phase I, ten patients were interviewed to identify the QoL domains impacted by chronic sialadenitis. In Phase II, these QoL domains were presented to a focus group of different chronic sialadenitis patients, who were asked to rank them by order of importance. A conceptual framework of QoL domains impacted by chronic sialadenitis was created based on patient consensus. Itemization of the PRO questionnaire was done by a focus group of four Otolaryngologists in phase III. Lastly, the questionnaire was completed in Phase IV by cognitive interviewing of five new chronic sialadenitis patients; ensuring ease of understanding and clarity.

**Results:**

Patients identified 15 domains of QoL impacted by chronic sialadenitis, divided into three sub-scales: physical symptoms, psychosocial symptoms, and activity restriction. These domains provided the basis for creation of a 22-item PRO questionnaire, with a Likert-type response scale.

**Conclusion:**

Clinical application of the novel questionnaire produced by this study will allow for a patient-centered assessment of the patient reported effectiveness of sialendoscopy assisted therapies for management of chronic sialadenitis.

*Level of evidence* Level V.

**Graphical Abstract:**

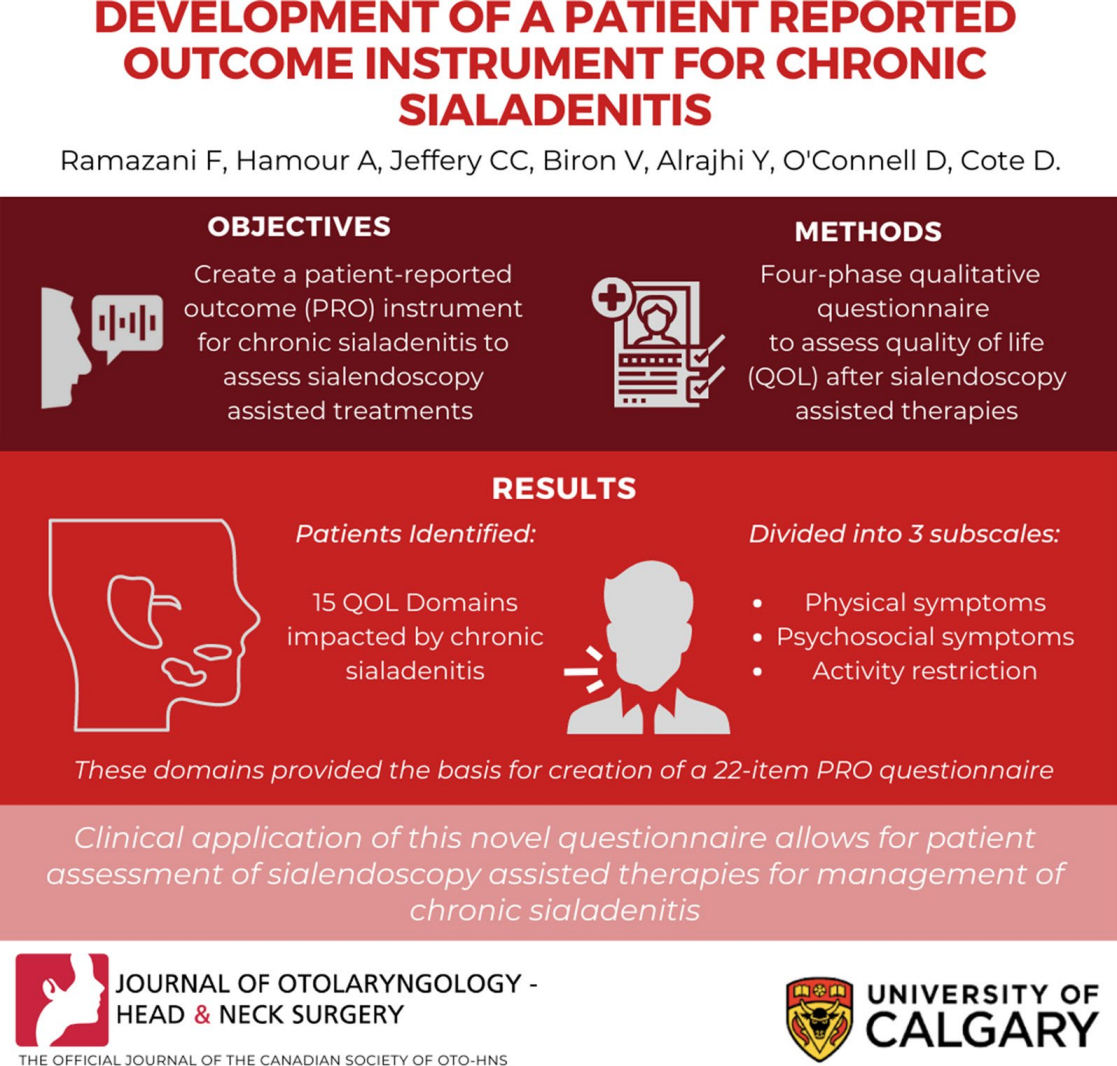

## Introduction

Chronic sialadenitis is a common benign condition of the salivary glands, affecting 1 in 20,000 individuals [1]. The most common underlying causes of this condition can be categorized into three groups: obstructive (e.g. sialolithiasis, foreign bodies, anatomic anomalies and ductal strictures), inflammatory (e.g. recurrent juvenile parotitis, and Sjogren’s syndrome), and iatrogenic (e.g. radiation exposure) [1–4]. Sialendoscopy is a technique used for intraluminal visualization of salivary ducts [5]. This technique is sometimes used in conjunction with instrumentation for management of underlying pathology—providing a gland preserving treatment option for patients with sialadenitis [3]. Use of sialendoscopy facilitated treatment of chronic sialadenitis has been associated with post-operative patient satisfaction and quality of life (QoL) improvement [2, 8–10]—providing symptomatic relief in up to 86% of treated patients [6, 7]. The current literature, however, has only made use of clinician or researcher designed instruments to assess patient satisfaction following sialendoscopy assisted treatment [7–10].

Patient reported outcomes (PROs) refer to reports coming directly from patients, without clinician interpretation, in regard to a given health condition [11]. The objective of this study was to create a comprehensive and patient centred instrument to assess the PROs of sialendoscopy assisted management of chronic sialadenitis.

## Materials and methods

### Study design and participants

Ethics approval was obtained from the Health Research Review Board (REB) at the University of Alberta (Pro00075887). This study took place at the University of Alberta (U of A) hospital in Edmonton, Alberta, Canada. Inclusion criteria for study participants included:

1. Adult patients (over the age of 18), diagnosed with chronic sialadenitis, and assessed at the U of A Otolaryngology clinic;
2. Staff Otolaryngologists with experience treating patients with chronic sialadenitis in their practice.

Patients who met inclusion criteria were identified from the principal investigator’s practice and recruited by phone. Random selection of patient participants was undertaken in all phases of this study, with no selection bias based on demographic or diagnostic information. A list of all patients in the principal investigator’s practice with a primary diagnosis of “sialadenitis” was identified and generated with no specific order (alphabetic, age, etc.). Patients were then selected from this list and contacted by phone for study recruitment. This process was undertaken for both Phase I and Phase IV of this study. Otolaryngologists were identified from the University of Alberta Division of Otolaryngology- Head and Neck Surgery (OHNS) staff database and recruited by email. All participants were consented as per REB guidelines.

This four-phase qualitative study employed grounded theory methodology and a modified Delphi technique. Grounded theory is an approach to qualitative research which explores and interprets varying viewpoints about a given topic, through the lens of various stakeholders [12]—in this case, patients with chronic sialadenitis and staff Otolaryngologists. This methodology aims to explore various interpretations of a concept; in this case the QoL implications of chronic sialadenitis. Employing grounded theory in research implies placing significant emphasis on data collection, and allowing theories to emerge from raw data, as opposed to using data to prove a pre-existing theory— it is therefore by definition an interpretive approach to research [12]. In this study, grounded theory allows for creation of a questionnaire based on the lived experiences and the common language used by patients with chronic sialadenitis. Furthermore, the integration of a modified Delphi technique allows for the triangulation of data by integrating both patient and physician perspectives, thus increasing the internal validity of the final questionnaire [13].

### Phase I—qualitative interviewing

The first phase of this study aimed to create a preliminary conceptual framework of the domains of life impacted by chronic sialadenitis. Patients were interviewed over the phone, and/or in person, and asked to describe the ways in which their life had been impacted by this condition. Interviews were conducted in a semi-structured manner by using open ended questioning to reduce any leading researcher biases [13]. Each interview was audio recorded and transcribed verbatim. Transcription of the interviews included both lingual and para-lingual components (intonations, laughter, pauses, etc.), allowing clarification of implicit meanings and relative importance of comments made by participants. Coding of the data was done in a line-by-line fashion using NVivo software. Line-by-line coding allowed for an organized approach to coding emergent themes in each interview. Each code was created to correspond to a potential quality of life domain impacted by chronic sialadenitis. In addition to line-by-line coding, conceptual “memo-ing” was undertaken for each interview. Through this process, the researcher was able to document their understanding of emergent themes in the data. Phase I was conducted using an alternating data collection and coding approach; each interview was transcribed and coded subsequent to its acquisition, prior to conducting the next patient interview. Following each additional interview, selective and axial coding allowed for core categorization and subcategorization of existing codes. The core categories corresponded to the domains of life most impacted by chronic sialadenitis. Questions asked in each subsequent interview were guided by the gaps in data identified by ongoing theoretical sampling, facilitated by selective coding. As a result, the questions in each interview became progressively less open ended and included more specific questions regarding areas which necessitated further elaboration. The patient interview and coding process continued until the point of theoretical saturation (n = 10 interviews), at which point no new codes were emerging from the data. The point of theoretical saturation was regarded as the endpoint of data collection, and the determinant of sample size for this first phase of the study. Following completion of all interviews in Phase I, core categories were used to create a conceptual framework, which was then carried forward to phase II.

### Phase II—conceptual framework ranking

This phase consisted of a patient focus group comprised of three new patient participants. Use of a small focus group for this phase allowed for in-depth discussion between participants—enabling a thorough appraisal of the conceptual framework created in phase I. During this in-person focus group, participants were asked to provide a ranking of the quality of life domains outlined in the conceptual framework created in phase I. Following individual rankings, participants were asked to clarify and explain their choices to other members of the group generating further discussion about the quality of life burden of chronic sialadenitis. Discussions were audio recorded and transcribed verbatim. The rankings and discussions from the focus group were used to refine the pre-existing conceptual framework from Phase I through ongoing selective and axial coding.

### Phase III—expert panel and PRO itemization

This phase of the study saw the presentation of the final conceptual framework from phase II to a panel of four expert Otolaryngologists. During this in-person focus group participants were asked to itemize a PRO questionnaire as well as an appropriate response scale. The discussion from this focus group was audio-recorded and transcribed verbatim. Following this discussion, a preliminary PRO questionnaire was created with a Likert-type response scale.

### Phase IV—cognitive interviewing

The final phase of the study consisted of in-person interviews with five new patients. Patients were asked to read the preliminary PRO questionnaire created in Phase III and provide verbal commentary on the quality of the questions (in terms of clarity, wording, and relevance). These interviews were audio recorded and transcribed verbatim. Appropriate modifications were made to the questionnaire following each interview. The sample size for this phase was determined by the point of data saturation, when no new changes were being recommended by participants.

## Results

Using the input of 18 patients with chronic sialadenitis and 4 staff Otolaryngologists, a 22-item PRO questionnaire was created to assess the QoL domains impacted by chronic sialadenitis. A total of 10 individual interview were conducted in Phase 1 (6 male, 4 female). Interviews in Phase I lasted between 10.5 to 31.4 min (mean of 17.8 min). One patient focus group was conducted in Phase II (3 female participants) lasting 1 h and 7 min. One staff Otolaryngologist focus group took place in Phase III (3 male, 1 female) lasting 1 h and 24 min. Phase IV consisted of five individual interviews (2 male, 3 female) with interview times ranging between 15.3 and 25.1 min (mean of 22.7 min).The age range of the 18 patient participants, across all four phases of this study, was between 23 and 68 (median age = 46). The underlying etiology of chronic sialadenitis in the study population was sialolithiasis for 14 participants (77.8%), Sjogren’s Syndrome for one patient (5.6%), and unknown for three patients (16.7%). Seventeen patients (94.4%) reported unilateral disease, while one patient (5.6%) reported bilateral chronic sialadenitis. The majority of patients in this study reported submandibular gland disease involvement (*N* = 12, 66.7%), compared to parotid involvement (*N* = 6, 33.3%).

A total of 26 core categories emerged from Phase I, corresponding to QoL domains impacted by chronic sialadenitis. Domains were included if they were mentioned by at least two participants as having significant impact on their quality of life. Following phase II, a final conceptual framework was created, using 15 patient-identified QoL domains. This framework can be separated into three subscales (Table 1), assessing the impact of physical symptoms as well as the psychosocial and activity restriction implications of living with chronic sialadenitis. The considerations involved in including a QoL domain in the framework included the number of times it was mentioned by participants, as well as the significance of the impact on QoL- based on both lingual and para-lingual features of patients’ descriptions. Tables 2, 3, and 4 provide details regarding the number of mentions of each domain, as well as illustrative quotes used in the coding process.

**Table 1:** Quality of life domains impacted by chronic sialadenitis, as reported by patient participants

Activity restriction subscale	Symptom subscale	Psychosocial subscale
Restricted eating	Swelling	Mood (frustration, irritability, and low mood)
Disrupted sleep	Pain	Self-consciousness about appearance
Time off occupation/studies/housework	Changes to Salivation (volume, taste, texture, and control of secretions)	Avoidance of social activities and social eating
	Difficulty chewing/Trismus	Anxiety
	Difficulty swallowing	Enjoyment of foods
	Palpable stones in mouth	
	Painful eating

**Table 2:** Patient reported physical symptoms associated with chronic sialadenitis

Physical symptoms	Number of participants *(N* = *10)*	Total mentions	Illustrative quote
Swelling	9	40	“There’s just so much swelling it looks like a deformity on the outside of my face.” -Interview 7
Pain	7	25	“I dealt with these bad sharp pains for quite a while” -Interview 2
Volume of Saliva	4	13	“The dry mouth is the only thing I’ve noticed.”- Interview 6
Texture of Saliva	2	3	“…my gland would swell up and then there would be a large discharge and a lot of sand and grit in my mouth…”- Interview 4
Taste of Saliva	2	5	“…it (saliva) would have a different taste. It was pretty salty, like a mineral, and sometimes it kind of had a metallic or mineral salt kind of taste.”-Interview 2
Drooling	4	8	“…it seems like I’m drooling more.” -Interview 5
Difficulty chewing/trismus	4	11	“I couldn’t open my jaw to the full capacity that I can usually open it, when I had the swelling” -Interview 8
Difficulty swallowing	3	5	“It just feels like the passageway isn’t fully open and that there is a blockage. Like it has to go around something it shouldn’t normally go around.” -Interview 6
Palpable stones in mouth	6	8	“I know it (stone) was always there because I could always feel it in my mouth.” -Interview 3
Painful eating	6	14	“It was a sharp pain, it felt like you were eating something sharp that stabbed you in the gums.” -Interview 2

**Table 3:** Patient reported psychosocial implications of chronic sialadenitis

Psychosocial symptoms	Number of participants *(N* = *10)*	Total mentions	Illustrative quote
Mood (frustration, irritability, and low mood)	5	12	“I wasn't happy” -Interview 3
			“it did effect my comfort, which effects your emotional state of mind.” -Interview 8
Self-consciousness about appearance	6	22	“I don’t like going out too much because people look at you weird.” -Interview 5
Avoidance of social activities and social eating	8	14	“I wouldn't go out very often because I couldn't eat, drink or anything.” -Interview 3
Anxiety	7	27	“I felt some anxiety (in anticipation of symptoms). Of course anxiety comes with frustration.” -Interview 7
Enjoyment of foods	3	9	“Recently last year or three years ago it started bothering me when I would eat something sour so that’s why it affected the type of food or the kind of food that I wanted to eat. I like sour stuff, even salt and pepper.” -Interview 10
			“A lot of times it would kill my appetite because I knew it was gonna be uncomfortable.” -Interview 8

**Table 4:** Patient reported activity restriction implications of chronic sialadenitis

Activity restriction domains	Number of participants *(N* = *10)*	Total mentions	Illustrative quote
Restricted eating	7	24	“I think I ate maybe one or two little things per day because I couldn't deal with anything else.” -Interview 3
Disrupted sleep	5	11	“It’s really really bad. It’s trouble falling asleep and waking up every couple of hours.” -Interview 7
Time off occupation/studies/housework	4	9	“Oh absolutely, yes when I get a flare up. I’ve missed work over it.” -Interview 7
			“…because it got uncomfortable and you wouldn’t want to go to class.” -Interview 8

In Phase III, the conceptual framework created based on patients’ lived experiences was used to itemize the final 22-item PRO questionnaire. During this phase, each domain was discussed by focus group participants until a consensus was reached regarding relevance, wording, and appropriate Likert-type response scales. Final modifications to wording and readability of the questionnaire were made in Phase IV following cognitive interviewing with five new patients.

## Discussion

As medicine moves to embrace a more patient-centred approach to care, it is important to create and employ outcome measures which incorporate the patient perspective. The reality of healthcare today is that practitioners’ limited patient interactions do not allow for adequate exploration of the QoL burden of medical conditions. In surgical specialties, this reality means that the effectiveness of surgical procedures in alleviating the QoL burden imposed by chronic conditions may not be fully understood or evaluated by physicians. As such, outcome measures solely created based on the opinion of healthcare practitioners or objective assessments (imaging, and investigations, etc.) may not always provide the most comprehensive assessment of an intervention’s effectiveness in improving patients’ QoL [14]. Patient reported outcome measures present a unique opportunity to assess the extent of patient satisfaction with surgical interventions [15]. This is particularly useful for new surgical procedures such as sialendoscopy, as PROs can contribute to a patient centred cost–benefit analysis for widespread use of a new procedure.

Chronic Sialadenitis, characterized by inflammation of the salivary glands, is most often managed with conservative measures or extirpation surgery. Sialendoscopy provides intraluminal visualization of salivary ducts and assistance with minimally invasive surgical management of this condition. Current sialendoscopy literature uses clinician or researcher designed instruments to assess the patient reported outcomes of these minimally invasive treatment modalities [6–8, 10]. While these studies have shown symptom improvement following interventions, it is not possible to assess whether the domains captured by these instruments encompass the breadth of disease burden imposed by chronic sialadenitis. By creating a PRO instrument grounded in patients’ experiences and input, this study addresses the lack of patient-centeredness in current sialadenitis outcome measures. The use of a qualitative approach in the study design ensured that the final questionnaire was one which was grounded in patient experiences, perspectives, and common language. Furthermore, integration of a modified Delphi methodology allowed for the triangulation of data between all primary stakeholders (patients and practitioners) involved in this procedure, thus increasing internal validity of the results [16]. Finally, cognitive interviewing with patients in phase IV enhanced respondent validation [17] by ensuring that patients found the PRO questionnaire clear, relevant, and easy to read.

The final questionnaire (Table 5) captures patients’ experiences with sialadenitis related symptoms, activity restriction, and psychosocial burdens of disease. Questions have been grouped into three distinct subscales (as outlined in Table 1), which will allow clinicians and researchers to identify which area has the greatest impact on patients’ quality of life. These subscales may also provide clinicians with the ability to direct patient care and counselling to the greatest areas of concern. While categorization of questionnaire items allows for enhanced interpretation of patient responses, the questionnaire itself is not itemized in sequence based on these subscales. The current sequence of items in the PRO allows for ease of readability and understanding, as per participant feedback in Phase IV. Following completion of the questionnaire, patients will receive a total score, as well as a score associated with each subscale (Table 1). Questionnaire items with a binary response scale will receive a score of 0 for responses indicating a lack of symptoms, and 1 for symptom presence. Questionnaire items with a four- or five-point Likert-type scale will receive scores ranging from 0 to 4 or 0 to 5, with 0 corresponding to responses indicating the absence of symptoms.

**Table 5:**
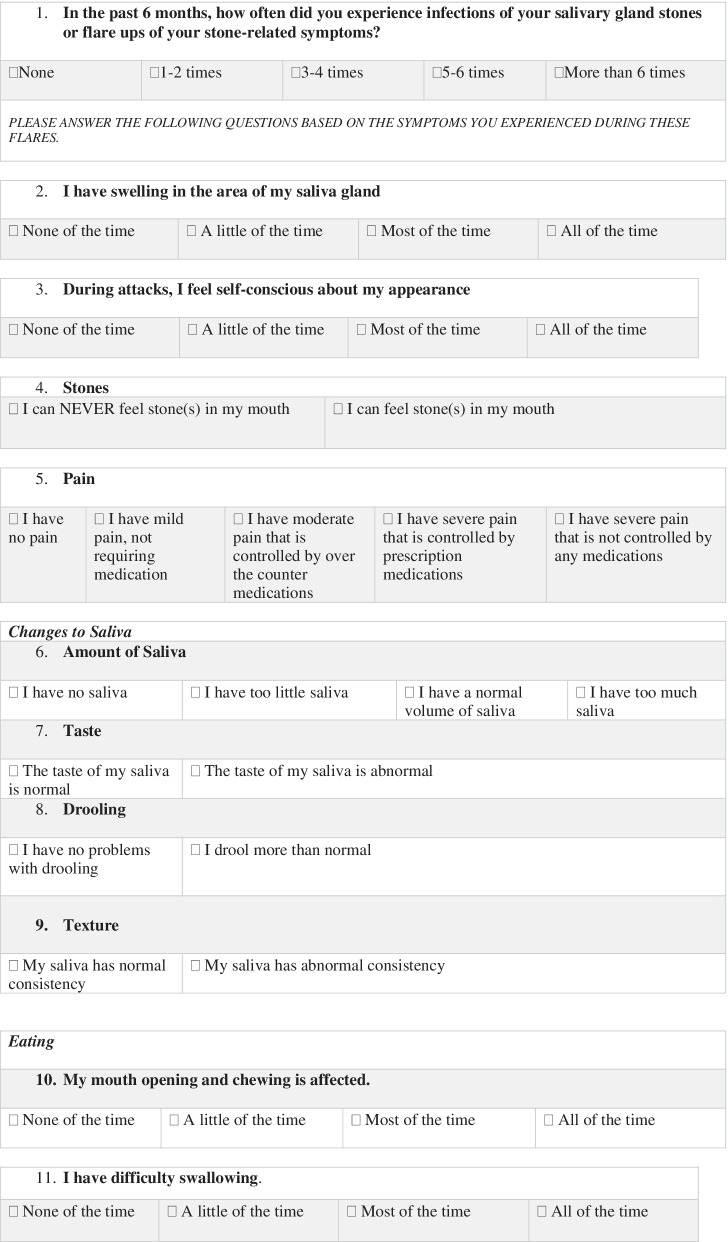

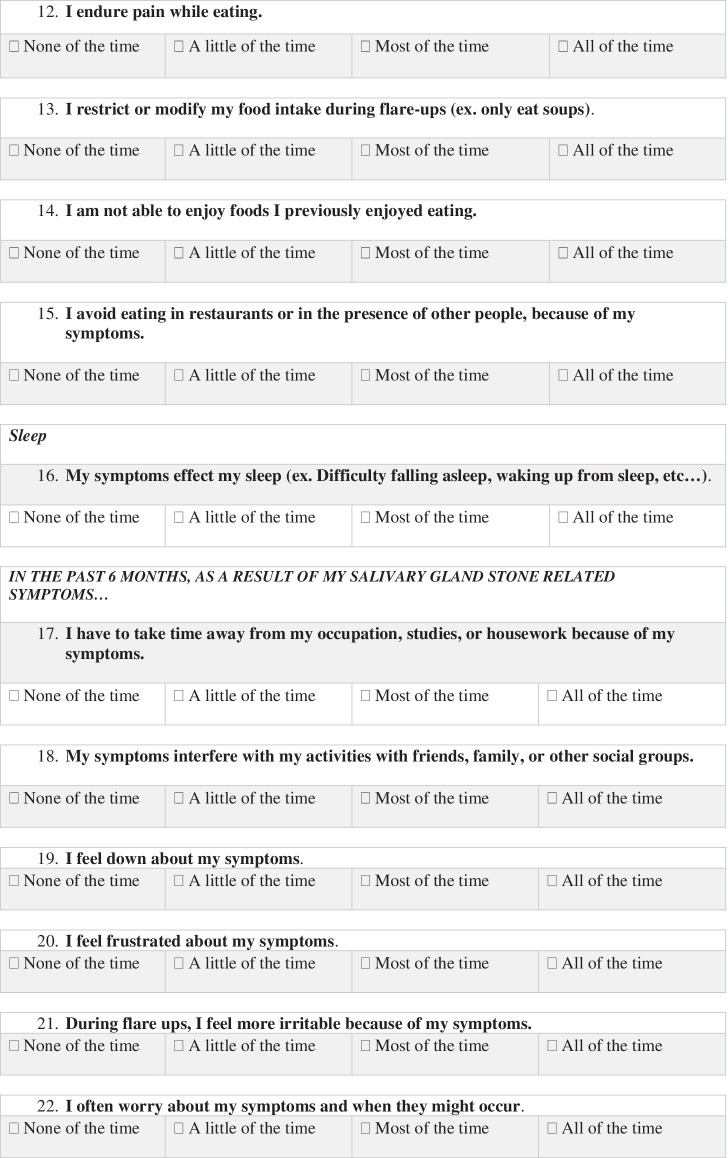
Patient reported outcomes questionnaire for sialendoscopy

Review of current literature reveals the use of clinician derived QoL questionnaires for assessment of the impact of chronic sialadenitis on patients’ lives, as well as treatment outcomes of sialendoscopy assisted procedures [6, 18]. Jokela et al. utilized the Health-Related Quality of Like instrument to assess the patient reported QoL benefits of Sialendoscopy assisted procedures [18]. While this 15-dimensional questionnaire is comprehensive in assessing various QoL domains, it encompasses symptoms (such as seeing and sexual activity) which are not conventionally associated with chronic sialadenitis and, based on the interviews conducted in this study, were not specifically mentioned by patients suffering from this chronic condition. Aubin-Pouliot et al. utilized the chronic obstructive sialadenitis symptoms (COSS) questionnaire to assess the impact QoL of sialendoscopy assisted procedures [6]. While this questionnaire focuses on sialadenitis specific symptoms, it was designed without the input of patients. Comparison of the COSS questionnaire with the PRO questionnaire described in this study reveals differences in wording and emphasis on certain QoL components (irritability, anxiety, and social interaction) which were selectively emphasized in our PRO questionnaire based on patient input and experiences. These differences highlight the importance of this study and directly incorporating patient experiences into the development of PRO instruments, as clinician perceived symptoms of concern may be different than those experienced by patients.

While this study’s contribution is unique in the field of sialadenitis and sialendoscopy, there remain some limitations to be explored. As with most qualitative studies, the issue of generalizability of this questionnaire remains to be tested^19^. While this tool is grounded in data generated from patient interviews, the opinions represent those of the patients sampled for this study. This does not account for potential differences in perception based on culture, age, or geography, with varying patient populations and a larger sample size. In future studies we plan to validate this instrument by administration to a large cohort of chronic sialadenitis patients. The prospective sample will include patients from varying demographic backgrounds and underlying etiology of disease (sialolithiasis, autoimmune disease, etc.…). Large scale validation will allow us to assess each item of the questionnaire based on the number of responses and nature of patient responses. Lastly, the potential for researcher bias proved challenging to mitigate in this study design, as the researcher is the primary instrument used to collect and analyze data. An attempt to lessen the potential for researcher bias was through the use of triangulation and various methods of data collection and interviewing—utilizing individual interviews, as well as focus groups.

## Conclusions

This study is the first of its kind to create a patient-centred PRO questionnaire to assess the effects of chronic sialadenitis on patients’ quality of life. This PRO allows for assessment of QoL domains which may otherwise not be explored in great depth in a clinical setting, due to competing demands imposed by limited time and high patient loads. As well, this novel outcome measure promises to be a valuable resource in assessing the effectiveness of sialendoscopy in alleviating the QoL burden of chronic sialadenitis. Future studies should aim to look at large scale implementation and external validation of this novel questionnaire in a clinical setting.

## Data Availability

The datasets during and/or analysed during the current study available from the corresponding author on reasonable request.
